# Effect of 21-Day Omega-3 Polyunsaturated Fatty Acid Supplementation on Exercise-Induced Secretory Factors and Inflammation Status in Young Men: A Randomized Double-Blind Trial

**DOI:** 10.3390/nu18030539

**Published:** 2026-02-06

**Authors:** Magdalena Konert, Paulina Brzezińska, Andrzej Kochanowicz, Elżbieta Piskorska, Błażej Stankiewicz, Ewa Polkowska, Tomasz Sledzinski, Adriana Mika, Jędrzej Antosiewicz, Jan Mieszkowski

**Affiliations:** 1 Department of Gymnastics and Dance, Faculty of Physical Education, Gdańsk University of Physical Education and Sport, 80-336 Gdańsk, Poland; magdalena.konert@awf.gda.pl; 2 Division of Clinical Physiotherapy, Faculty of Physical Education, Gdansk University of Physical Education and Sport, 80-336 Gdańsk, Poland; paulina.brzezinska@awf.gda.pl; 3 Faculty of Health Sciences, University of Lomza, 18-400 Łomża, Poland; andrzej.kochanowicz@awf.gda.pl (A.K.); epolkowska@al.edu.pl (E.P.); 4 Department of Pathobiochemistry and Clinical Chemistry, Nicolaus Copernicus University Collegium Medicum, 85-094 Bydgoszcz, Poland; piskorska_e@cm.umk.pl; 5 Department of Biomedical Basis of Physical Education, Faculty of Health Sciences and Physical Education, Kazimierz Wielki University, 85-091 Bydgoszcz, Poland; blasta@ukw.edu.pl; 6 Department of Pharmaceutical Biochemistry, Medical University of Gdańsk, 80-211 Gdańsk, Poland; tomasz.sledzinski@gumed.edu.pl (T.S.); adriana.mika@gumed.edu.pl (A.M.); 7 Department of Bioenergetics and Physiology of Exercise, Medical University of Gdańsk, 80-211 Gdańsk, Poland

**Keywords:** muscle damage, inflammation status, *n*-3 polyunsaturated fatty acids, *n*-6 polyunsaturated fatty acids, physical performance

## Abstract

**Objectives**: The objective of this study was to evaluate the effect of 21-day dietary omega-3 fatty acid supplementation on the levels of postexercise inflammation response, oxidative stress, and selected exerkine secretion among physically active young men. **Methods**: In a randomized double-blind study, 24 physically active men were assigned to two groups: a supplementation group (n = 12), receiving 3250 mg of *n*-3 polyunsaturated fatty acids (PUFAs) daily, and a placebo group (n = 12). Blood samples were collected before and after twenty-one days of dietary supplementation to measure total fatty acids and inflammatory markers, including IL-1β, IL-6, IL-10, BDNF, and FGF23. **Results**: After 21 days of *n*-3 fatty acid supplementation, there were no significant changes in anaerobic performance parameters. However, significant interactions were found in the systemic immune-inflammation index (SII), FGF-23, IL-1β, IL-1Ra, IL-6, and IL-10 in response to exercise and supplementation. **Conclusions**: 21 days of *n*-3 fatty acid supplementation modified PUFA content and influenced inflammation status, but did not affect maximal anaerobic performance.

## 1. Introduction

Highly intense physical exercise and athletic performance lead to exercise-induced muscle damage and increase the production of reactive oxygen species (ROS), resulting in oxidative stress, greater muscle inflammation, decreased strength, muscle soreness, and a potentially increased risk of injuries in the future [1,2,3,4].

Increased ROS production—which accompanies the postexercise inflammatory response—is associated with increased inflammatory cytokine secretion in the circulatory system due to muscle damage [5]. Despite numerous studies investigating the effects of anaerobic exercise on oxidative stress and inflammatory reactions, the results of applied models for attenuating this type of response are debatable, highlighting a significant research gap in effective strategies for reducing exercise-induced inflammation [2,6,7,8,9,10,11,12]. Beyond classical inflammatory cytokines, acute exercise also stimulates the release of circulating secretory factors often referred to as exerkines, which contribute to systemic signaling and recovery-related processes. Nevertheless, it remains unclear whether short-term *n*-3 fatty acid supplementation can modulate the acute exerkine response to high-intensity anaerobic exercise.

It has been suggested that antioxidant supplementation can alleviate oxidative stress and acute inflammatory responses induced by exercise. Polyunsaturated (*n*-3) fatty acids (PUFAs) are essential nutrients that must be obtained through diet, as the body cannot produce them internally. The shortest of these, alpha-linolenic acid (ALA; 18:3), is commonly found in seeds and nuts. In contrast, eicosapentaenoic acid (EPA; 20:5) and docosahexaenoic acid (DHA; 22:6) are longer-chain *n*-3 PUFAs typically sourced from marine foods, such as fatty fish and seals. While much of the research on *n*-3 fatty acid supplementation has centered on their potential and beneficial effects on various inflammation-related conditions, such as cardiac dysrhythmias, rheumatological diseases, and lipid dysregulation [13,14], increasing scientific evidence suggests that they may also benefit neural functions [15] and support exercise adaptations [16,17,18]. Moreover, it has been proven that manipulating the omega-3 polyunsaturated fatty acid (PUFA) content of skeletal muscle may improve muscle function, metabolism [19,20] and have the potential to enhance muscle regeneration by positively modulating the local and systemic inflammatory response to muscle injury (e.g., increased M2 and decreased M1 macrophages after 2 and 3 days of muscle injury) [21].

Additionally, supplementation with polyunsaturated (*n*-3) fatty acids may inhibit lipid peroxidation and interfere with the arachidonic acid cascade by inhibiting 5-Lox and leukotriene B4 (LTB4), consequently lowering LTB4 production [22]. Therefore, the consumption of omega-3 fatty acids may ameliorate the negative effects of intense exercise and the subsequent inflammatory response.

This study aims to assess the effects of a 21-day omega-3 fatty acid supplementation period on the secretion of selected exerkines and inflammation markers following acute high-intensity anaerobic exercise in physically active young men. The choice of a 21-day supplementation period is based on previous research indicating that this type of short-duration supplementation period is sufficient to observe physiological changes and induce adaptations in response to omega-3 fatty acid intake, making it a relevant timeframe for evaluating its short-term impacts on exercise recovery and inflammation [23,24].

## 2. Materials and Methods

### 2.1. Experimental Overview

The study was structured as a double-blind, randomized controlled trial featuring parallel groups. Participants underwent a 21-day daily supplementation regimen, receiving either 3250 mg of *n*-3 polyunsaturated fatty acids (PUFAs) or a placebo. Before and following this supplementation period, participants engaged in maximal anaerobic efforts, specifically two anaerobic Wingate tests (WAnTs). To assess the impact of maximal anaerobic exertion and supplementation on the participants’ inflammatory states, venous blood samples were collected for serum analysis at designated time points, both prior to and following supplementation, in accordance with the protocols approved by the European Federation of Clinical Chemistry and Laboratory Medicine.

In addition, all markers selected for analysis were evaluated according to the medical diagnostic standardized procedures referenced by the European Federation of Clinical Chemistry and Laboratory Medicine (https://www.eflm.eu/) [25,26].

**Figure 1:**
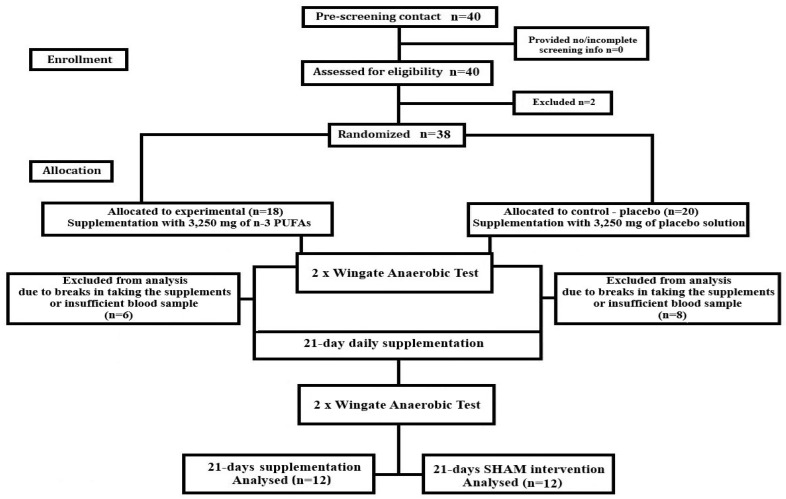
Consort flow diagram of study procedures.

At the outset of the study, participants’ ages, body compositions, and heights were recorded. They were also acquainted with the testing equipment and procedures. All laboratory analyses took place at the University of Physical Education and Sport (GUPES) and the Medical University in Gdansk, adhering to the protocols specified in the CONSORT flow diagram (Figure 1).

### 2.2. Participants

Thirty-eight physically active, healthy young men were enrolled in the study. Participants were randomly divided into two groups: an experimental (supplementation, n = 18) group and a control (placebo, n = 20) group. All participants were examined by a professional physician and found to be healthy. Due to some virial or bacterial infections experienced during the supplementation period, minor injuries that prevented participants from completing the exercise trials, interruptions in supplement intake, and the inability to participate in follow-up exercise trials or insufficient blood sample amounts undermining the identification of statistically significant changes in serum omega-3, 24 participants finally completed the experiment (supplementation group, 12 participants aged 22.00 ± 1.41 years, and control group, 12 participants aged 21.81 ± 2.04 years). The participants’ descriptive physical characteristics are presented in Table 1.

All the participants declared that they engaged in regular recreational sports (running, swimming, or team sports) but not more than two to three times per week for 45 min each time. The participants had maintained good health over the past six months and had no bone or muscle tissue injuries or medical histories of cardiovascular, autonomic nervous system, immunological, or other diseases that could potentially influence the study results.

During the experiment, as well as for three months preceding it, the participants took no drugs and had not suffered from minor injuries or health problems. They were all informed about the study procedures prior to enrollment, but they were not aware of the study’s aims or the supplementation schedule.

### 2.3. Supplementation

Both groups successfully completed a 21-day daily supplementation program. The choice of a 21-day supplementation period is based on previous research indicating that this type of short-duration supplementation period is sufficient to observe physiological changes and induce adaptations in response to omega-3 fatty acid intake, making it a relevant timeframe for evaluating its short-term impacts on exercise recovery and inflammation [23,24].

The supplementation group consumed 5000 mg of omega-3 PUFA-rich oil in gelatin capsules (Nutropharma, Lesznowola, Poland) containing 3250 mg of *n*-3 PUFAs, including 1650 mg of EPA and 1100 mg of DHA, along with 500 mg of other *n*-3 PUFAs. In contrast, the placebo group consumed equal numbers of visually identical gelatin capsules filled with a water-based solution. Rigorous gas chromatography–mass spectrometry (GC–MS) analyses of the supplements confirmed that the active compounds, their compositions, and quantities matched the manufacturer’s specifications.

Regardless of whether they were placed in the *n*-3 PUFAs or the placebo group, everyone received gelatin capsules of the same color, flavor, size, and smell, packaged in identical black containers with a barcode on both the lid and the side wall of the container. Each participant was required to swallow the supplements and drink water, which left no real chance of the capsule opening and its contents escaping. This prevented the samples from being decoded at any stage of the study.

During the supplementation period, all participants received capsules three times, for each 7-day period. Each time, patients received 35 capsules (105 capsules for the whole period), and we measured adherence (count-backs) according to: adherence (%) = 100 × D(D − R), where D = number of capsules issued to the participant, and R = capsules returned by the participant.

Only blood samples from participants with 100% adherence were included in the final research analyses, meaning that participants took all the capsules they were given.

### 2.4. Dietary and Physical Activity Control

During the two weeks prior to the experiment, all participants were asked to maintain their usual diet and physical activity. Two weeks prior to the experiment, they were given a 48 h food control protocol and asked to record all the foods and drinks they consumed to ensure nutrition homogeneity.

Two days prior to the end of the trial (on days 20–21 of supplementation), the participants completed a second 48 h food control protocol under the control of a professional dietician. Mobile electronic reminders were also provided regularly throughout the experiment to ensure the consistency of supplementation, diet, and exercise practices throughout the study. Food control was conducted to allow for the quantification of total energy intake and macronutrient (carbohydrate, protein, fatty acid) and omega fatty acid consumption before and during the supplementation period.

In addition, to avoid increasing polysaturated omega-3 fatty acids before the start of the supplementation period and to exclude people that consumed statistically larger amounts of omega-3 fatty acids than the rest of the population, all participants were provided with a list of foods rich in omega-3 fatty acids that could not be eaten during the experiment and specific data for the ALA and EPA/DHA was collected.

According to the collected data, all participants were consuming a similar amount, around 1.38 g/daily of ALA and 164.20 mg/daily total of EPA/DHA.

Participants’ fluid intake was carefully controlled and recorded throughout all stages of the study. Additionally, hydration levels were assessed directly at the time of testing, ensuring that any potential effects of dehydration on metabolism and performance were minimized.

### 2.5. Maximal Anaerobic Effort

The Bar-Or (1987) [27] protocol was followed to assess maximal anaerobic effort using WAnTs on a cycle ergometer (Monark 894E, Peak Bike, Sweden). The WAnTs were repeated twice to ensure accurate results. During each test, the participant’s saddle height was adjusted individually.

Prior to testing, all participants underwent a standardized warm-up session on the cycle ergometer (5 min at 60 rpm, 1 W/kg).

Subsequently, during the WAnT testing phase, each individual was instructed to pedal vigorously with maximum effort for a 30 s period against a fixed resistive load of 75 g/kg of total body mass. A brief 30 s pause followed the initial WAnT before the second WAnT was conducted under the same conditions as during the first WAnT. All achieved parameters were recorded on a personal computer using MCE 5.1 JBA Zb Software (Staniak, Poland) [28].

The following WAnT variables were measured: peak power (W) and relative peak power (W/kg), calculated as the highest single point of power output (recorded at 0.2 s intervals); and mean power (W) and relative mean power (W/kg), calculated as the average power output during the 30 s test.

### 2.6. Sample Collection and Measurement of n-3 PUFA Levels

Blood samples were collected at four time points before and after 21 days of *n*-3 fatty acid supplementation: I, before; II, immediately after; III, 6 h after; and IV, 24 h after the 2 × 30s WAnTs. The same procedure was repeated after the 21-day supplementation period and the second 2 × 30s WAnT analysis.

The blood was collected in 5 mL BD Vacutainer Clot Activator Tubes (Becton Dickinson Co., Franklin Lakes, NJ, USA). The serum was separated by centrifugation at 4000 g for 10 min and aliquoted into 500 μL portions according to the medical diagnostic standardized procedures referenced by the European Federation of Clinical Chemistry and Laboratory Medicine. The samples were frozen and stored (for no longer than 6 months) at −80 °C until further analysis.

Total lipids were extracted from whole serum samples in a chloroform–methanol mixture (2:1, *v*/*v*) as previously described [29]. Fatty acids were determined from the resulting total serum lipid extract. Lipid extracts were dried by evaporation under a stream of nitrogen. Each sample was hydrolyzed with 1 mL of 0.5 M KOH in methanol at 90 °C for 3 h. The mixture was acidified with 0.2 mL of 6 M HCl, and 1 mL of water was then added. The non-esterified fatty acids were extracted three times with 1 mL of n-hexane and evaporated until dry in a nitrogen stream. Fatty acid methyl esters (FAMEs) were prepared with 1 mL of a 10% boron trifluoride-methanol solution at 55 °C for 90 min. One mL of water was added to the reaction mixture, the FAMEs were extracted three times with 1 mL of n-hexane, and the solvent was evaporated. The FAMEs were analyzed using GC-EI-MS QP-2010 SE (Shimadzu Corporation, Kyoto, Japan). They were separated on a 30 m 0.25 mm i.d. Rtx-5MS capillary column (film thickness 0.25 μm). The column temperature was set at 60–300 °C at a rate of 4 °C/min with helium as the carrier gas and a column head pressure of 60 kPa. For FAME ionization, the electron energy was 70 eV, and the internal standard was 19-methylarachidic acid. Data are presented as percentages (% of total identified fatty acids), calculated by normalizing each GC peak area to the sum of all identified fatty acids. Total fatty acids (*n*-3 and *n*-6) were observed before and 24 h after the 2 × 30s WAnTs (before and after the supplementation period).

Levels of the following inflammatory response and other serum markers were determined: brain-derived neurotrophic factor (BDNF), fibroblast growth factor (FGF-23), interleukin 1β (IL-1 β), interleukin 1 receptor antagonist (IL-1 Ra), interleukin-6 (IL-6), interleukin-10 (IL-10) and resistin The analyses were conducted using a MAGPIX fluorescence detection system (Luminex Corp., Austin, TX, USA) with Luminex assays [Luminex Corp.; Luminex Human Magnetic Assay]. For selected proteins, microspheres were used, each one excited by red (635 nm) and green (525 nm) LEDs and classified according to the different amounts of fluorescence emission depending on the analyte to be studied. Plates (96-well) were incubated with the antigen for binding to the capture antibody of each microsphere. After incubation, the biotinylated detection antibody for each marker was added. Finally, a streptavidin–phycoerythrin complex (Strep-PE) was added to bind the detection antibody. The fluorescence of all samples was measured, and, according to a received standard curve, the analysis program calculated the concentration of each marker of interest using the mean fluorescence intensity (MFI).

An accredited laboratory (Synevo Laboratory, Warsaw, Poland; PN-EN ISO 15189 [30]) analyzed the blood samples to determine the values of hematological parameters using flow cytometry on a Sysmex XS-1000i apparatus (Sysmex Corporation, Kobe, Japan), a hematological analyzer, and an immunoenzymatic method. From the obtained data, changes in the SII index were calculated.

In the past years, the calculated systemic immune-inflammation index (SII) has gained increasing recognition due to its reliable insight into the inflammatory responses, especially in the case of basic morphological diagnostics in different populations. It was calculated as [platelet [PLT] × absolute neutrophil [NEU]/absolute lymphocyte [LY] count)/1000] using the hematological parameters before and immediately after the 2 × 30s WAnTs (before and after the 21-day supplementation period) [31].

### 2.7. Statistical Analysis

Descriptive statistics included the means ± standard deviations (SDs) for all measured variables. The normality of distribution was checked using a Shapiro–Wilk’s test, and Levene’s test was used to check the homogeneity of variance.

To evaluate changes in selected inflammatory marker levels before and after the 2 × 30s WAnTs, a two-way (2 × 4) repeated measures (RM) analysis of variance (ANOVA) was performed, where group (GR) was the between-subject factor (supplementation or control) and the RMs were the within-subject factors (I, II, III, and IV). Differences in body composition and maximal anaerobic performance during the first and second WAnTs were evaluated using two-way (2 × 2) repeated measures ANOVAs with the same GR and RM factors (before and after supplementation). To control for multiplicity arising from testing multiple outcomes, a Bonferroni correction was applied to the *p*-values of the primary ANOVA main effects and interaction terms (including the GR × RM interaction).

The effect sizes of the participants’ characteristics and selected markers were determined using eta-squared statistics (ƞ2): ƞ2 values ≥ 0.01, 0.06, and 0.14 were the threshold values for small, moderate, and large effect sizes, respectively. A power analysis was performed using GPower v. 3.1.9.2 for the interactions between the effects to determine the appropriate sample size [32]. Accordingly, for a medium effect size and test power of 0.80, the minimum required sample size was 24 participants.

All graphics were generated using GraphPad Prism 6.0 (GraphPad Software, Boston, MA, USA). All calculations were made using Statistica 12 (StatSoft, Tulsa, OK, USA). Differences were considered statistically significant at *p* ≤ 0.05.

### 2.8. Ethics

The study was conducted according to the guidelines of the Declaration of Helsinki, and approved by the Bioethics Committee for Clinical Research at the Collegium Medicum Nicolaus Copernicus in Torun (protocol code. KB 536/2017; with the first date of approval: 17 June 2017) and further modifications approved by the Bioethics Committee for Clinical Research at the Regional Medical Chamber in Gdansk (consent No. KB23/22; from 9 May 2022). Study was registered in ClinicalTrials.gov with ID NCT05520437 (30 August 2022 as a first trial registration date).

Prior to the commencement of the study, all the participants provided written informed consent. They were all provided with detailed information about the study’s objectives and testing procedures, as well as the option to withdraw their consent at any point for any reason.

## 3. Results

The percentage composition of total *n*-3 and *n*-6 PUFAs at baseline and after twenty-one days of dietary supplementation is presented in Table 2. As a secondary outcome, the relative abundance of serum *n*-3 PUFAs increased significantly from baseline to day 21 in the omega-3 supplementation group (+140.1%, *p* < 0.01). Moreover, after 21 days, serum *n*-3 PUFA values were significantly higher in the omega-3 supplementation group than in the placebo group (189.7%; *p* < 0.01). In contrast, the relative abundance of *n*-6 PUFAs showed no meaningful change over time in either group (Table 2).

The characteristics of maximal anaerobic performance before and after 21 days of *n*-3 PUFA supplementation are presented in Table 3. As another secondary outcome, the ANOVA showed no significant changes in anaerobic performance across the parameters after twenty-one days of dietary supplementation.

**Figure 2:**
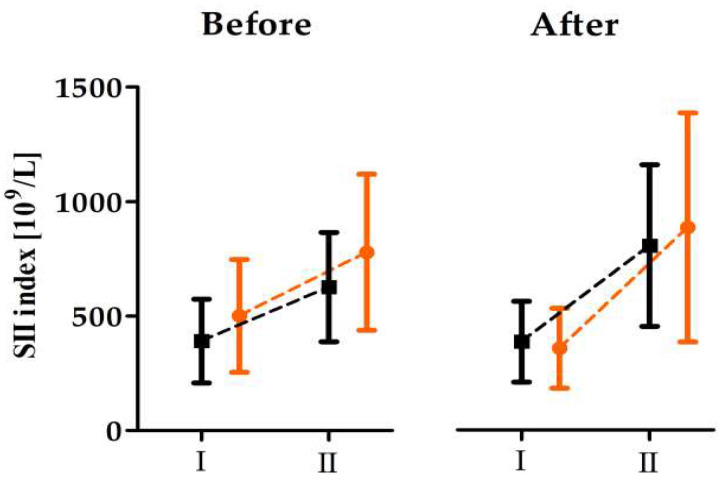
Changes in the SII index induced by 2 × 30-s WAnTs before and after twenty-one days of dietary supplementation with omega-3 or a placebo (mean + SD). **Note:** Orange indicates the supplementation group (n = 12) and black indicates the placebo group (n = 12); I, before the 2 × 30s WAnTs; II, immediately after the 2 × 30s WAnTs.

The baseline hematological values for the evaluated parameters did not differ between the groups. Nevertheless, in response to anaerobic exercise, a two-way ANOVA showed, both before and after twenty-one days of dietary supplementation, significant increases in WBC (before, 34.5%, *p* < 0.01; after, 45.7%, *p* < 0.01) NEU (before, 64.8%, *p* < 0.01; after, 94.9%, *p* < 0.01), and MCHC (before, 1.0%, *p* < 0.01; after, 1.2%, *p* < 0.01), and a decrease in HCT immediately after 2 × 30-s WAnTs (before, 2.7%, *p* < 0.05; after, 5.3%, *p* < 0.01) (Table 4). Moreover, significant increases in the SII index were also noticed directly after the 2 × 30s WAnTs before and after omega-3 supplementation (Figure 2).

Regarding the primary outcomes (inflammatory and exerkine markers), two-way ANOVA baseline values for the selected inflammatory parameters showed significant changes in BDNF, IL-beta, and IL-10 after 21 days of omega-3 supplementation. In the omega-3 supplementation group, the serum concentrations of BDNF increased significantly (+16.4%, *p* < 0.05) and IL-10 (+31.6%, *p* < 0.01), but the concentration of IL-1β decreased significantly (−21.3%, *p* < 0.05). The analysis and serum inflammation marker changes induced by the 2 × 30s WAnTs before and after twenty-one days of dietary supplementation with omega-3 or a placebo are presented in Table 5. A significant effect of WAnTs was recorded for BDNF, IL-1β, IL-1 Ra, IL-10, and resistin both before and after twenty-one days of dietary supplementation. Regardless of the group factor, significant increases in BDNF (before, +36.1%, *p* < 0.01; after, +25.2%, *p* < 0.01), IL-1β (before, +48.9%, *p* < 0.01; after, +31.6%, *p* < 0.01), IL-1 Ra (before, +67.8%, *p* < 0.01; after, +25,2%, *p* < 0.05) IL-10 (before, +17.3%, *p* < 0.05; after, +17.1%, *p* < 0.01), and resistin concentrations (before, 52.6%, *p* < 0.01; after, 31.3%, *p* < 0.01) were observed immediately after the 2 × 30s WAnTs compared to the baseline values. FGF-23 (before, 32.2%, *p* < 0.01) and IL-6 (before, 49.7%, *p* < 0.01) showed a significant WAnT effect only before 21 days of omega-3 supplementation.

**Figure 3:**
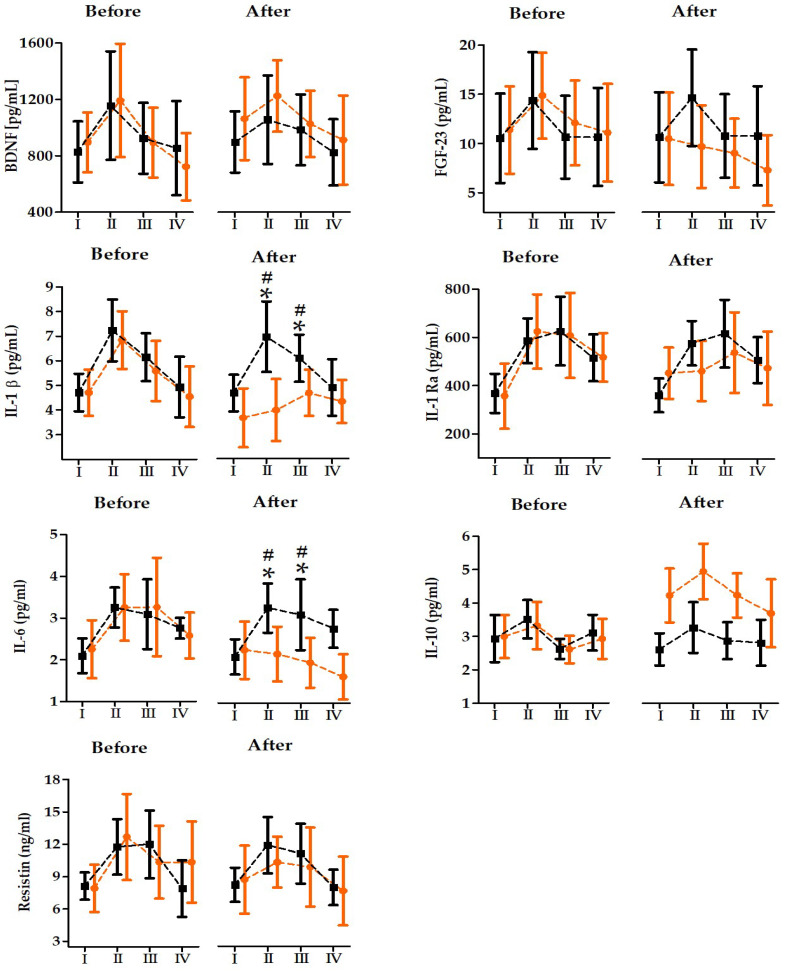
Changes in serum inflammation levels induced by 2 × 30-s WAnTs before and after twenty-one days of dietary supplementation with omega-3 or a placebo (mean ± SD). **Note:** Orange indicates the omega-3 supplementation group (n = 12); black indicates the placebo group (n = 12); I, before; II, immediately after; III, 6 h after; and IV, 24 h after the 2 × 30s WAnTs. * Significant difference vs. the other group; # significant difference vs. placebo group before the 2 × 30-s WAnTs. The significance level was set at Bonferroni-adjusted *p* < 0.01.

A significant group × time interaction was detected after 21 days of omega-3 supplementation for IL-1β and IL-6 (Table 5; Figure 3), indicating differential post-exercise responses between groups after supplementation. Post hoc results showed an attenuated response in the omega-3 group, as significant immediate post-exercise increases were observed in the placebo group for IL-1β (+53.2%, *p* < 0.01), IL-1Ra (60.1%, *p* < 0.01), and IL-6 (55.0%, *p* < 0.01), whereas the corresponding changes were not significant in the supplementation group (Figure 3).

Accordingly, between-group comparisons revealed lower concentrations in the omega-3 group immediately after the WAnTs for IL-1β (−46.1%, *p* < 0.01) and IL-6 (−34.0%, *p* < 0.01), and at 6 h post-exercise for IL-1β (−22.9%, *p* < 0.05) and IL-6 (−36.6%, *p* < 0.01) (Figure 3). In contrast, IL-10 showed a significant main effect of group, indicating higher serum concentrations in the omega-3 group than in placebo across all time points after 21 days of supplementation (overall +44.7%, *p* < 0.01).

## 4. Discussion

This study demonstrates that three weeks of omega-3 PUFA supplementation can modify PUFA content and blunt several exercise-induced inflammatory responses after repeated supramaximal Wingate efforts, without improving maximal anaerobic performance. From an applied sport science perspective, these data are most consistent with a recovery-supporting role of omega-3 intake during congested training or competition schedules, while highlighting that performance outcomes likely depend on longer exposure and sport-specific loading conditions.

High-intensity strenuous exercise is widely known to induce muscle damage and inflammation, affecting many aspects of an athlete’s body functioning [5,33,34,35]. However, regular physical activity and physical training induce adaptations that contribute to the body’s higher resistance to exercise-induced inflammation and oxidative changes [36,37].

Unfortunately, in many situations, the capability to attenuate postexercise inflammation through adaptation-dependent mechanisms is not sufficient for proper body functioning during extremely demanding exercise [37]. Hence, researchers, trainers, and sports specialists have sought ways to improve the body’s inflammation resistance. A well-known and widely used approach involves the consumption of antioxidant-rich products, often used as “emergency” strategies to mitigate acute postexercise oxidative and inflammatory stress [38,39,40]. In contrast to classical antioxidant rescue approaches, *n*-3 PUFAs may modulate exercise-induced inflammation through changes in membrane fatty acid composition and downstream lipid-mediated inflammatory signaling, rather than acting as direct antioxidants [41,42]. In view of the preceding considerations, the aim of this study was to investigate the effects of short-term (21-day) *n*-3 fatty acid supplementation on circulating intense-exercise-induced secretory factors and inflammation in physically active young men. The most important finding of this study is that three weeks of omega-3 PUFA supplementation blunted the acute post-exercise responses of the key proinflammatory cytokines IL-1β and IL-6 after repeated supramaximal Wingate efforts. For IL-1Ra and FGF-23, exercise-related changes were observed; however, after multiplicity adjustment, evidence for a differential group-specific post-exercise response should be interpreted with caution. In contrast, IL-10 was overall higher in the omega-3 group across measurement time points after supplementation.

While most studies have focused on the effects of aerobic exercise on immunological homeostasis and inflammation, only limited evidence exists concerning the effects of anaerobic exercise on immune system activity [5,33]. Thus far, studies have shown that eccentric resistance exercise increases total WBC counts, suggesting a similar effect for aerobic exercise [43,44,45].

In this context, a significant level of muscle damage induced by demanding exercise may trigger a strong immune response. Regarding the previous considerations, an increase in WBC indicated that a 2 × 30s WAnT significantly affected the immune balance. However, 21-day supplementation with *n*-3 PUFAs effectively attenuated white blood cells, lymphocytes, monocytes, and neutrophils, suggesting reduced postexercise immune system activation. Szymanska et al. [25] drew similar conclusions, indicating that an anti-inflammatory diet rich in omega-3 fatty acids could contribute to a decrease in immune response, hematological markers, and affect the SII index. However, relatively little research has focused on direct *n*-3 fatty acid supplementation and changes in the SII index in response to supramaximal anaerobic exercise; therefore, our work adds novel evidence by combining a randomized double-blind design with SII assessment, inflammatory cytokines, and selected exerkines measured in response to repeated 2 × 30s WAnTs.

Few researchers have assessed oxidative stress after intense exercise only through hematological changes, since hematological analyses should be used in combination with serum/plasma markers to provide more reliable evidence of the magnitude of muscle damage. Hence, researchers have focused on sample evaluations combined with immunoenzymatic analyses to indirectly reveal selected secretory and inflammation marker responses [46]. In the present study, we focused mainly on changes in brain-derived neurotrophic factor, fibroblast growth factor, resistin, FGF-23, IL-1 β, IL-1 Ra, IL-6, and IL-10.

During and after intense exercise, interactions between immune cells, cytokines, and other intracellular components create an inflammatory milieu that promotes recovery from and adaptation to exercise [47,48,49]. Additionally, physical exercise induces muscle damage and a nonspecific inflammatory response, which manifests in elevated concentrations of circulating proinflammatory cytokines, such as IL-1 β, TNF-α, and IL-6 [50,51].

The main cytokines responsible for regulating the inflammatory process are IL-1 and IL-6, which play important roles in initiating and regulating the postexercise inflammatory process. Rising levels of those cytokines are the most common changes observed in most athletes and nonathletes after physical activity [50,52]. Furthermore, IL-6 is the first proinflammatory cytokine to increase after exercise and is the main mediator of the effects of exercise [53]. IL-6 also plays a metabolic role during exercise, as its increased levels stimulate fatty acid mobilization. Importantly, IL-6 may be released from multiple sources, including contracting skeletal muscle, where it acts as a myokine, as well as immune cells, which supports its pleiotropic metabolic and immunomodulatory roles after intense exercise [54,55,56]. We measured the overall IL-6 changes that reflect the body’s response to physical stress.

Both cytokines—IL-1 and IL-6—showed a WAnT-dependent reaction. In the case of the control group, the observed increase reached statistical significance, especially before and after the supplementation period. Other studies showed that post-exercise increases in IL-6 stimulated the appearance of other anti-inflammatory cytokines, such as IL-1ra and IL-10 [57,58]. The current study revealed similar changes in IL-6 levels immediately after exercise and before the supplementation period. After 21 days of omega-3 supplementation in the supplementation group, 2 × 30s WAnTs did not affect post-exercise IL-6 changes. In the control group, the observed changes provided evidence of induced inflammation. This attenuation of IL-6 in the supplementation group may be partly explained by the concomitant increase in IL-10, which can contribute to the resolution of inflammation and limit excessive cytokine amplification, although this interpretation should be treated as a plausible mechanism rather than a confirmed pathway. Importantly, transient elevations in proinflammatory cytokines are not inherently detrimental because they contribute to immune cell trafficking, tissue remodeling, and subsequent adaptation. Therefore, the observed attenuation should be interpreted as a potential fine-tuning of the acute inflammatory response rather than an unequivocal “suppression” that is always beneficial.

IL-1ra exerts a similar anti-inflammatory activity to IL-10, mainly by blocking IL-1 receptors and thereby preventing proinflammatory IL-1 signal transduction [59]. For the IL-1 receptor antagonist, we observed similar increasing trends in both groups. However, the control group showed a numerically larger post-exercise increase. This difference can be attributed to the fact that the precise nature of the relationship between IL-6 and IL-1ra is still largely unknown. It may be that, during physical activity, the local muscle production of IL-6 participates in the systemic induction of IL-1ra changes in circulating blood mononuclear cells. Hence, a larger postexercise increase in IL-6 may be associated with greater IL-1ra changes, and these responses may be interrelated [60,61,62].

Resistin is a proinflammatory cytokine secreted by various tissues, predominantly by immune system cells such as lymphocytes, monocytes, and macrophages in humans [63]. Discovered in 2001, this protein was initially linked to obesity and type 2 diabetes, indicating its role in insulin resistance, which is reflected in its name “resistin” [64]. Current research has revealed that resistin serves multiple other functions. Beyond influencing insulin sensitivity, it plays a role in inflammation and inflammatory diseases, including atherosclerosis and arthritis, regulates carbohydrate and lipid metabolism, and promotes the proliferation of endothelial cells [65]. Studies have shown a positive correlation between circulating levels of resistin and common inflammatory and fibrinolytic biomarkers like CRP, TNF-α, and IL-6 in conditions such as type 2 diabetes, rheumatoid arthritis, chronic kidney disease, sepsis, and coronary atherosclerosis [66]. Notably, engaging in regular physical activity has been shown to significantly reduce resistin levels [67]. However, during extreme physical exertion—such as marathons or ultra-marathons—levels of inflammatory markers tend to increase [5]. Our research showed that serum resistin levels increased similarly in both groups in response to physical activity, aligning with reports from Vella et al. [67] and Mieszkowski et al. [5] who revealed the post-exercise response of this exerkine. In the current study, 21-day omega-3 supplementation did not affect the increase in exercise-induced resistin levels in physically active young men. The direct effect of omega-3 on postexercise-induced resistin levels has not been widely studied and requires further exploration. However, increases in resistin levels during exercise appear to limit exercise capacity, and the observed lack of any statistically significant post-supplementation resistin changes, along with decreasing trends, should be considered a favorable change [5].

BDNF—a neuronal protein in the central nervous system—plays an important role in brain functioning, which directly depends on the developmental stage of life [68]. BDNF can affect metabolic processes such as energy homeostasis, glucose metabolism regulation, appetite, and many other body functions [68,69]. A meta-regression analysis proved that changes in BDNF are physical-activity-dependent, and greater durations of exercise are associated with greater increases in BDNF [70]. Hence, in our study, brain-derived neurotrophic factor reached statistical significance in response to acute exercise (2 × 30s WAnT), but this effect did not differ between the supplemented and control groups, and we did not observe supplementation-related changes. It seems that such short-term dietary manipulation may not affect BDNF secretion. Only a much longer period of dietary manipulation (e.g., for 8 weeks) is likely to lead to dietary-dependent BDNF changes [71]. However, the analysis of the literature on the relationship between BDNF and omega-3 supplementation leads to the conclusion that the connection between omega-3 fatty acids and BDNF levels may be influenced by various factors. One such pathway, for example, can be attributed to the activation of BDNF transcription by the DHA molecule through the PI3K/Akt signaling pathway [72]. Consequently, although an increase can be achieved, it was not evident following short-term supplementation in this study.

FGF23, also known as fibroblast growth factor 23, is a risk factor for cardiovascular mortality in individuals with chronic kidney disease [63]. Interestingly, the consumption of *n*-3 PUFAs may have an inverse correlation with FGF23 levels and cardiovascular risk [73]. Previous data suggest that *n*-3 PUFAs may influence FGF-23 dynamics [74]. In the present study, FGF-23 responded to repeated supramaximal exercise; however, after Bonferroni correction for multiple testing, the group × time interaction did not remain significant, and any supplementation-related modulation of FGF-23 should be interpreted cautiously. Future prospective studies are warranted to clarify whether *n*-3 PUFAs, particularly at lower doses or with shorter supplementation periods, and whether exercise modality, meaningfully affect FGF-23 responses.

Overall, the presented findings support the hypothesis that 21-day supplementation with 5000 mg omega-3 PUFA-rich oil containing 3250 mg of *n*-3 PUFAs may attenuate proinflammatory cytokine and secretory protein excretion, which will take place without significant changes in exercise capacity. While our study did not yield significant performance enhancements, the modulation of inflammatory responses through *n*-3 fatty acid supplementation offers a promising strategy for athletes aiming to manage post-exercise inflammation, especially in the case of direct competition.

Significant changes in cytokine levels were revealed, with a decrease in interleukin-1 (IL-1b) and interleukin-6 (IL-6), and an increase in interleukin-10 (IL-10) after twenty-one days of dietary supplementation with 3250 mg of *n*-3 polyunsaturated fatty acids (PUFAs) suggesting that *n*-3 PUFAs may play a crucial role in modulating cytokine secretion, especially in the context of physical exertion. Eicosapentaenoic acid and docosahexaenoic acid are known to incorporate into cell membranes, impacting membrane fluidity and the function of membrane-bound receptors and enzymes. This incorporation can influence intracellular signaling pathways and may be one of the main reasons for the observed changes in the cytokine production [75]. Moreover, during intensive physical exercise, tissues such as muscles and immune cells release cytokines in response to stress and micro-injuries. *n*-3 PUFAs can downregulate the activation of nuclear factor-kappa B (NF-κB), a transcription factor that promotes the expression of pro-inflammatory cytokines like IL-1 and IL-6 [76]. This inhibition leads to a reduced inflammatory response. Additionally, *n*-3 PUFAs can enhance the activity of peroxisome proliferator-activated receptors (PPARs), which play a role in the transcription of IL-10, fostering an environment conducive to inflammation resolution [77]. Furthermore, by altering the balance of eicosanoid production from arachidonic acid to more anti-inflammatory mediators, *n*-3 PUFAs reduce the synthesis of pro-inflammatory agents and increase the production of resolvins and protectins, which aid in resolving inflammation [42].

These findings underscore the potential of targeted nutritional interventions to facilitate recovery and may enhance training frequency under conditions of high-intensity exercise. Moreover, speculative interpretations regarding the relationship between omega-3 intake and specific cytokine pathways should be explicitly categorized as hypotheses. There is a growing body of evidence supporting the beneficial roles of pro-inflammatory cytokines in muscle repair and regeneration following exercise, warranting further inquiry in subsequent studies. Mechanistic links between omega-3 intake and specific cytokine pathways should be interpreted as plausible mechanisms and explicitly framed as hypotheses when causal inference is not directly supported by the study design.

The presented research has many strengths, including the relatively high dose of supplementation (5000 mg of omega-3 PUFA-rich oil), which is very rarely obtained through diet, that was given to the participants. Moreover, analyses of a highly homogenous male population, with directly restricted inclusion/exclusion criteria and dietary management confirms the reliability of the obtained results, eliminating the risk of random trends in the observed changes. Furthermore, supramaximal physical effort (2 × 30s WAnT) is an extremely demanding type of physical activity that is associated with many induced biochemical changes at various stages of body functioning, which significantly burdens the body’s immune system, inducing a number of metabolic and biochemical changes

### Limitations

As mentioned previously, the present study has some limitations. The research did not follow a dose–response design. Moreover, during the examinations, we did not measure other parameters that could affect the oxidative state, exercise hormonal balance, vitamin D, and other factors [5]. The aforementioned dose of omega-3 can be treated as both a strength and a limitation due to the fact that the dose of *n*-3 PUFA supplementation was relatively high and sufficient to change the fatty acid composition of cholesteryl esters. Furthermore, in Western Europe, it is difficult to maintain such a high nutritional dose of omega-3, so this type of experiment can be repeated only under synthetic laboratory supplementation conditions. Additionally, the relatively high participant dropout (omega-3 supplementation group completion rate = 66.67%, sham control = 60%) may have influenced our results. Finally, the analyzed population was relatively small, which limited the collection of blood samples due to the exclusions during the examinations and research. A small sample size could have influenced the results, although differential associations with outcomes have been calculated for small samples. Furthermore, due to the limited amount of blood samples, we did not measure lipid mediators such as resolvins, protectins, and maresins, nor did we investigate whether omega-3 supplementation reduces pro-inflammatory eicosanoids. Such observations could provide additional insights into the mechanisms by which omega-3 fatty acids exert their effects on inflammation status.

It is important to emphasize that one-directional supplementation, even among athletes, focusing solely on omega-3 fatty acids, may not always be fully beneficial due to metabolic changes in their biotransformation and other PUFAs biotransformation [78].

It is crucial to balance the diet with omega-6 and omega-9 fatty acids as well. A holistic approach to fatty acid intake can help ensure optimal health and performance, as each type of fatty acid plays a distinct role in the body’s metabolic processes. Therefore, a well-rounded dietary strategy that includes a variety of fatty acids is essential for achieving the best outcomes.

## 5. Conclusions

**Figure 4:**
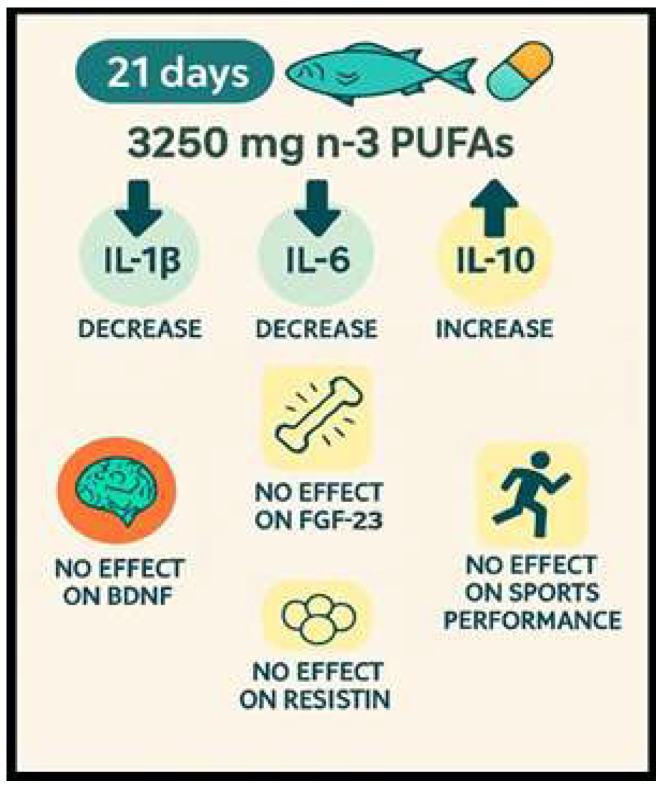
Main effect of 21 days of n-3 PUFA supplementation protocol.

This study demonstrated that twenty-one days of dietary supplementation with 3250 mg of *n*-3 PUFAs altered PUFA content and was associated with an attenuated post-exercise inflammatory response to repeated 2 × 30 s Wingate efforts, primarily reflected by reduced IL-1β and IL-6 responses and higher overall IL-10 concentrations (Figure 4). However, this supplementation did not enhance maximal anaerobic performance and did not affect FGF-23, resistin and BDNF.

Future research should further explore the long-term effects of *n*-3 PUFA supplementation on health and physical performance, especially with longer intervention periods, dose–response designs, and the integration of additional serum markers (e.g., oxidative stress indices, endocrine responses, muscle damage markers), combined with analyses of muscle biopsies and gene expression to clarify the pathways through which omega-3 PUFAs exert their effects.

## Figures and Tables

**Table 1:** Descriptive physical characteristics before and after twenty-one days of dietary supplementation.

Variable	Unit	Placebo Group (n = 12)		Omega-3 Supplementation Group (n = 12)	
		Before	After	Before	After
		Mean ± SD	Mean ± SD	Mean ± SD	Mean ± SD
Body height	cm	181.22 ± 8.10	-	179.14 ± 7.36	-
Body mass	kg	79.88 ± 7.84	79.36 ± 7.26	77.67 ± 7.10	76.03 ± 6.77
Body fat percentage	%	12.78 ± 5.11	12.43 ± 4.97	12.03 ± 4.56	11.50 ± 4.31
Body mass index	kg/m^2^	23.98 ± 3.68	23.67 ± 3.56	23.51 ± 3.23	23.76 ± 3.05

**Table 2:** Descriptive baseline levels of *n*-3 and *n*-6 PUFAs before and after twenty-one days of dietary supplementation with omega-3.

Variable	Unit	Placebo Group		Omega-3 Supplementation Group	
		Before	After	Before	After
		Mean ± SD	Mean ± SD	Mean ± SD	Mean ± SD
*n*-3 PUFAs	%	2.45 ± 0.72	2.52 ± 1.13	3.04 ± 0.39	7.30 ± 1.51 *†
*n*-6 PUFAs	%	33.50 ± 2.73	32.52 ± 4.90	35.35 ± 2.92	34.18 ± 2.47

* Significant difference versus baseline (Before) within the omega-3 supplementation group (*p* < 0.01). † Significant between-group difference at day 21 (After) for *n*-3 PUFAs (*p* < 0.01).

**Table 3:** Characteristics of maximal anaerobic performance before and after twenty-one days of dietary supplementation (mean ± standard deviation [SD]).

Variable	Unit	Placebo Group		Omega-3 Supplementation Group	
		Before	After	Before	After
		Mean ± SD	Mean ± SD	Mean ± SD	Mean ± SD
Absolute peak power (1st WAnT)	W	789.91 ± 127.63	737.89 ± 161.85	831.76 ± 127.63	825.95 ± 212.38
Absolute peak power (2nd WAnT)	W	557.38 ± 100.53	543.70 ± 79.65	542.81 ± 111.73	553.45 ± 103.54
Absolute mean power (1st WAnT)	W	593.40 ± 81.87	541.33 ± 179.78	620.21 ± 111.04	607.05 ± 120.18
Absolute mean power (2nd WAnT)	W	410.21 ± 67.22	413.11 ± 72.91	384.05 ± 44.54	389.66 ± 43.88
Relative peak power (1st WAnT)	W/kg	10.09 ± 1.32	9.44 ± 1.75	9.86 ± 1.14	9.72 ± 1.04
Relative peak power (2nd WAnT)	W/kg	7.29 ± 0.99	6.99 ± 0.96	6.46 ± 0.76	6.58 ± 0.69
Relative mean power (1st WAnT)	W/kg	7.59 ± 0.89	6.95 ± 2.16	7.40 ± 0.68	7.20 ± 0.65
Relative mean power (2nd WAnT)	W/kg	5.24 ± 0.73	5.33 ± 0.97	4.68 ± 0.83	4.72 ± 0.76

**Note:** WAnT = Wingate anaerobic test.

**Table 4:** Descriptive values for changes in hematological parameters induced by 2 × 30s WAnT s before and after 21 days of omega-3 supplementation.

Variable	Unit	Placebo Group (n = 12)				Omega-3 Supplementation Group (n = 12)			
		Before Twenty-One Days of Dietary Supplementation with Omega-3		After Twenty-One Days of Dietary Supplementation with Omega-3		Before Twenty-One Days of Dietary Supplementation with Omega-3		After Twenty-One Days of Dietary Supplementation with Omega-3	
		Baseline	Immediately After 2 × 30s WAnTs	Baseline	Immediately After 2 × 30s WAnTs	Baseline	Immediately After 2 × 30s WAnTs	Baseline	Immediately After 2 × 30s WAnTs
		Mean ± SD	Mean ± SD	Mean ± SD	Mean ± SD	Mean ± SD	Mean ± SD	Mean ± SD	Mean ± SD
WBC	10^9^/L	6.95 ± 2.31	9.33 ± 3.31	6.44 ± 2.19	9.36 ± 2.44	6.97 ± 1.22	9.40 ± 1.70	5.98 ± 0.98	8.21 ± 1.44
LY	10^9^/L	2.42 ± 0.79	2.33 ± 0.64	2.23 ± 0.70	2.11 ± 0.68	2.18 ± 0.60	2.48 ± 0.77	2.05 ± 0.52	1.77 ± 0.38
MO	10^9^/L	0.66 ± 0.24	0.74 ± 0.16	0.68 ± 0.22	0.75 ± 0.27	0.72 ± 0.15	0.65 ± 0.27	0.65 ± 0.18	0.70 ± 0.21
NEU	10^9^/L	3.72 ± 1.66	6.14 ± 1.60	3.45 ± 1.50	6.46 ± 2.01	3.82 ± 1.28	6.07 ± 2.63	2.68 ± 0.66	5.64 ± 1.82
RBC	10^12^/L	4.85 ± 0.26	4.89 ± 0.23	4.76 ± 0.32	4.94 ± 0.37	4.89 ± 0.23	4.85 ± 0.26	4.80 ± 0.23	4.90 ± 0.33
HGB	g·dL^−1^	14.34 ± 0.95	14.51 ± 0.29	15.0.1 ± 0.85	14.45 ± 0.87	14.51 ± 0.64	14.34 ± 0.98	14.72 ± 0.81	14.24 ± 0.68
HCT	%	41.02 ± 2.24	40.63 ± 0.92	41.16 ± 2.31	39.93 ± 2.53	41.72 ± 1.14	40.05 ± 2.77	42.22 ± 1.93	40.13 ± 1.69
MCV	fL	84.64 ± 3.44	84.77 ± 4.26	84.76 ± 2.90	84. 74 ± 3.04	85.38 ± 4.23	84.10 ± 3.23	86.64 ± 4.4	85.25 ± 4.23
MCH	pg	30.02 ± 1.30	30.26 ± 1.52	30.13 ± 1.20	30.13 ± 1.16	30.14 ± 1.54	30.12 ± 1.36	30.04 ± 1.33	30.03 ± 1.38
MCHC	g·dL^−1^	35.48 ± 0.77	35.71 ± 0.50	35.38 ± 0.68	35.90 ± 0.69	35.32 ± 0.45	35.81 ± 0.85	35.02 ± 0.63	35.50 ± 0.53
PLT	10^9^/L	249.26 ± 36.02	248.77 ± 31.04	245.53 ± 34.38	245.44 ± 27.89	260.33 ± 40.55	278.31 ± 47.89	244.11 ± 23.48	245.26 ± 37.26
MPV	fL	11.10 ± 0.99	10.66 ± 0.84	11.02 ± 0.79	11.09 ± 1.03	10.71 ± 0.94	10.76 ± 0.79	10.81 ± 0.76	10.76 ± 0.71
RDW	%	12.95 ± 0.47	13.02 ± 0.64	12.70 ± 0.34	12.62 ± 0.33	13.15 ± 0.63	12.87 ± 0.48	12.73 ± 0.56	12.60 ± 0.52

**Note:** WBC = white blood cells, LY = lymphocytes, MO = monocytes, NEU = neutrocytes, RBC = red blood cells, HGB = hemoglobin, HCT = hematocrit, MCV = mean corpuscular volume, MCH = mean corpuscular hemoglobin, MCHC = mean concentration of corpuscular hemoglobin concentration, PLT = platelets, MPV = mean platelet volume, RDW = red blood cell distribution width.

**Table 5:** Two-way ANOVA (2 groups × 4 RMs) values for changes in serum inflammation marker levels induced by 2 × 30s WAnTs before and after twenty-one days of dietary supplementation with omega-3 or a placebo.

Variable		Effect	F	*Df*	*p*	Effect Size (η^2^)	Post Hoc Outcome
BDNF	Pre	GR RM GR × RM	0.02 16.48 1.15	1, 20 3, 60 3, 60	0.88 <0.01 * 0.33	0.01 0.45 0.05	II > I, III, IV
	Post	GR RM GR × RM	1.13 15.15 1.43	1, 25 3, 75 3, 75	0.29 <0.01 * 0.24	0.05 0.43 0.07	II > I, III, IV
FGF-23	Pre	GR RM GR × RM	0.20 14.01 0.23	1, 25 3, 75 3, 75	0.65 <0.01 * 0.86	0.01 0.41 0.01	II > I, III, IV
	Post	GR RM GR × RM	2.24 4.52 2.78	1, 25 3, 75 3, 75	0.13 <0.01 * 0.05	0.11 0.18 0.13	II > IV
IL-1β	Pre	GR RM GR × RM	1.04 29.30 0.33	1, 25 3, 75 3, 75	0.32 <0.01 * 0.80	0.05 0.59 0.01	II > III > I, IV
	Post	GR RM GR × RM	36.93 8.07 6.83	1, 25 3, 75 3, 75	<0.01 * <0.01 * <0.01 *	0.64 0.28 0.25	S > P II, III > I PII > PI, PIV; SII < PII
IL-1 Ra	Pre	GR RM GR × RM	0.01 28.17 0.31	1, 25 3, 75 3, 75	0.94 <0.01 * 0.081	<0.01 0.58 0.02	II, III > I < IV
	Post	GR RM GR × RM	0.47 8.97 3.65	1, 25 3, 75 3, 75	0.49 <0.01 * 0.02	0.02 0.31 0.15	II, III > I, IV
IL-6	Pre	GR RM GR × RM	0.06 10.75 0.28	1, 25 3, 75 3, 75	0.80 <0.01 * 0.83	<0.01 0.34 0.01	II, III > I, IV
	Post	GR RM GR × RM	51.12 4.45 10.02	1, 25 3, 75 3, 75	<0.01 * <0.01 * <0.01 *	0.22 0.70 0.11	S < P II > IV PII, PIII > PI, SII, SIII; SI < SIV
IL-10	Pre	GR RM GR × RM	0.26 7.69 0.27	1, 25 3, 75 3, 75	0.61 <0.01 * 0.84	0.01 0.27 0.01	II > I, III
	Post	GR RM GR × RM	56.75 9.28 3.00	1, 25 3, 75 3, 75	<0.01 * <0.01 * 0.03	0.73 0.31 0.13	S > P II > I, III, IV
Resistin	Pre	GR RM GR × RM	0.28 10.97 1.56	1, 25 3, 75 3, 75	0.59 <0.01 * 0.20	0.01 0.35 0.07	II, III > I
	Post	GR RM GR × RM	0.38 11.56 1.00	1, 25 3, 75 3, 75	0.54 <0.01 * 0.39	0.01 0.37 0.05	II, III > I, IV

**Note:** BDNF = brain-derived neurotrophic factor, FGF-23 = fibroblast growth factor, IL-1β = interleukin 1β, IL-1 Ra = interleukin 1 receptor antagonist, IL-6 = interleukin 6, IL-10 = interleukin 10. **Study design:** GR = group factor, RM = repeated measurement factor, P = placebo group; S = omega-3 supplementation group (n = 12); I, before; II, immediately after; III, 6 h after; and IV, 24 h after the 2 × 30s WAnTs. Significant effects are indicated at * Bonferroni-adjusted *p* ≤ 0.01.

## Data Availability

The data that support the findings of this study are available from the corresponding authors, [J.M., J.A.] upon reasonable request.

## Abbreviations

The following abbreviations are used in this manuscript:

SII index	systemic immune-inflammation index
2 × WAnT	two-time anaerobic Wingate tests
IL-1β	interleukin 1β
IL-1	interleukin 1
IL-6	interleukin 6
IL-10	interleukin 10
BDNF	brain-derived neurotrophic factor
FGF-23	fibroblast growth factor
PGE2	prostaglandin E2
TxA_2_	thromboxane A_2_
CysLTs	cysteinyl leukotrienes
LTB_4_	leukotriene B_4_
12(S)-HETE	12(S)-hydroxyeicosatetraenoic acid
20-HETE	20-hydroxyeicosatetraenoic acid
GC–MS	gas chromatography–mass spectrometry
